# BARRIERS AND STRATEGIES TO ENGAGE AND RETAIN DIVERSE RURAL DYADS IN PSYCHOSOCIAL INTERVENTIONS

**DOI:** 10.1093/geroni/igac059.3128

**Published:** 2022-12-20

**Authors:** Michael McCarthy, Y Evie Garcia, Mara Cassady, Neshay Mall, Pamela Bosch, Steven Barger

**Affiliations:** Northern Arizona University, Flagstaff, Arizona, United States; Northern Arizona University, Flagstaff, Arizona, United States; Northern Arizona University, Flagstaff, Arizona, United States; Northern Arizona University, Flagstaff, Arizona, United States; Northern Arizona University, Flagstaff, Arizona, United States; Northern Arizona University, Flagstaff, Arizona, United States

## Abstract

It is often challenging to engage and retain rural Latinx and Native Americans in psychosocial interventions after stroke, despite the fact that these populations tend to experience poorer health outcomes. Although strategies for effective patient-provider communication exist, few studies focus on the cultural aspects of engagement and retention of diverse dyads in a rural context. The aim of this study is to describe barriers to engagement and retention of diverse rural dyads in post-stroke psychosocial interventions, as well as to elucidate specific strategies to address these barriers. Semi-structured interviews were conducted with Nf14 clinical providers with extensive experience serving diverse rural couples and families. Qualitative data were analyzed using interpretive description methods. Seven key barriers were identified, grouped broadly into: 1. Those presented by providers (1a. Lack of time and attention given to dyads “telling their story”; 1b. Lack of culturally appropriate interpersonal skills; 1c. Incongruence between provider-patient values) and 2. Those existing within dyads themselves (2a. Low priority given to interpersonal and emotional issues; 2b. Concern about confidentiality in small communities and mistrust of those in power due to current or historical experiences with dominant cultures; 2c. Perception of stigma surrounding interpersonal and emotional issues; 2d. Tendency to be polite, accommodating, or feign investment). Providers offered specific culturally-informed strategies to overcome barriers which in turn may increase engagement and minimize attrition. Providers and researchers may benefit from these insights as they work to communicate with, build trust, and meet the unique needs of these populations.

